# Moderate Reduction in Dietary Protein Improves Muscle Composition and Modulates Gut Microbiota and Serum Metabolome Without Compromising Growth in Finishing Pigs

**DOI:** 10.3390/ani15223234

**Published:** 2025-11-07

**Authors:** Tengfei He, Zirong Ye, Chengwan Zhou, Songyu Jiang, Linfang Yang, Yanzhi Liu, Shunqi Liu, Jianfeng Zhao, Shenfei Long, Zhaohui Chen

**Affiliations:** 1 State Key Laboratory of Animal Nutrition and Feeding, College of Animal Science and Technology, China Agricultural University, Beijing 100193, China; hetengfei@cau.edu.cn (T.H.); 14715593589@163.com (Z.Y.); chengwan@cau.edu.cn (C.Z.); jsy18345753075@cau.edu.cn (S.J.); liusq57@cau.edu.cn (S.L.); zhaojianfeng@cau.edu.cn (J.Z.); 2 Guangdong Yihao Local Pig Research Institute Co., Ltd., Zhanjiang 524000, China; animal2302@163.com (L.Y.); 13997987819@163.com (Y.L.); 3 Beijing Jingwa Agriculture Science and Technology Innovation Center, Beijing 101205, China

**Keywords:** low-protein diet, meat quality, muscle composition, gut microbiota, serum metabolomics

## Abstract

**Simple Summary:**

Reducing dietary protein is an effective way to decrease nitrogen emissions and feed costs in pig production, but concerns remain about whether this affects growth performance and meat quality. This study examined how low-protein diets (LPDs) affect growth, meat quality, gut microbiota, and metabolism in finishing pigs. Results showed that moderate protein reduction, when balanced with essential amino acids, maintained growth and nutrient digestibility. However, protein levels influenced muscle fatty acid composition and amino acid profiles. LPDs also reshaped gut microbiota and serum metabolites, indicating adjustments in lipid and amino acid metabolism. Overall, moderate protein reduction can improve meat quality and gut health while reducing nitrogen output, offering a sustainable nutritional strategy for swine production.

**Abstract:**

Reducing dietary crude protein (CP) while sustaining growth performance and minimizing nitrogen emissions is a critical challenge in swine production. Beyond growth efficiency, the influence of low-protein diets (LPDs) on meat quality traits, gut microbiota, and systemic metabolism in finishing pigs remains insufficiently understood. In this study, 180 healthy crossbred finishing pigs (Duroc × Liangguang Small Spotted; initial body weight 85.49 ± 4.90 kg) were assigned to three dietary regimens for 35 days (six replicate pens per treatment, ten pigs per pen, male/female = 1:1): Control (CON, 15.5% CP), Low-Protein 1 (LP1, 14.5% CP), and Low-Protein 2 (LP2, 13.5% CP). Growth performance and nutrient digestibility were not impaired by protein reduction. Notably, LP1 pigs exhibited thicker backfat (*p* < 0.05), while LP2 pigs showed decreased concentrations of specific fatty acids (C12:0–C22:1n9) and essential amino acids (aspartic acid, glutamic acid, lysine) compared with LP1 (*p* < 0.05), indicating that dietary protein levels affected muscle composition. Cecal microbiota analysis revealed distinct shifts, with *Prevotella* spp., *Faecalibacterium* spp., and *Plesiomonas* spp. enriched in CON, whereas LP1 promoted *Eubacteriaceae* spp., *Christensenellaceae* spp., and *Clostridia* spp. (*p* < 0.05). Serum metabolomics further distinguished groups: LP1 reduced bile secretion and cholesterol metabolism pathways (*p* < 0.05) and LP2 further suppressed cholesterol metabolism and primary bile acid biosynthesis (*p* < 0.05), with a trend toward reduced phenylalanine metabolism (*p* = 0.07). Collectively, these findings demonstrate that moderate dietary protein reduction, when balanced with essential amino acids, maintains growth, reduces nitrogen output, and beneficially alters muscle composition, gut microbiota, and host metabolic pathways, offering nutritional strategies to enhance pork quality and promote sustainable pig production.

## 1. Introduction

The global livestock industry is under increasing pressure to reduce its environmental footprint, particularly regarding nitrogen emissions from pig production [1]. Over 70% of pig feed comprises nitrogenous compounds, of which incomplete digestion leads to elevated nitrogen levels in feces [2]. The subsequent volatilization of nitrogen as ammonia contributes significantly to atmospheric pollution [3]. To address these challenges, low-protein diets (LPDs), also known as ideal amino acid diets, have emerged as a promising strategy to reduce nitrogen excretion while maintaining growth performance [4,5]. LPDs are designed to meet the specific amino acid requirements of animals at different growth stages by adjusting the types, quantities, and proportions of dietary amino acids. The concept of low-protein diets has been widely adopted in pigs, poultry, and other livestock species [6,7]. Compared to the dietary protein levels recommended by the National Research Council (NRC) in 1998 [8], the NRC 2012 guidelines [9] suggest a reduction in crude protein (CP) by 2–4% in pig diets. This highlights the considerable potential and advantages of LPDs in the swine industry. LPDs present a cost-effective alternative by reducing the cost of protein-rich feed ingredients while maintaining performance parameters, including growth rate, carcass characteristics, and meat quality [10]. Furthermore, previous studies have shown that for every 1% decrease in dietary protein, total nitrogen excretion can be reduced by 8–10%, underscoring the value of LPDs as an environmentally friendly feeding strategy to mitigate nitrogen emissions and environmental pollution [11,12].

Although previous research has mainly focused on the effects of LPDs on growth performance and nitrogen balance, the systemic effects of protein restriction—particularly on meat quality, gut microbiota, and host metabolism—remain insufficiently explored [13,14]. LPDs can alter the composition of the intestinal microbiota, potentially enriching bacterial taxa involved in nitrogen cycling, such as *Christensenellaceae* [15]. However, the integrative impact of protein level reduction on the gut microbiota, host metabolic pathways, and muscle nutrient profiles in finishing pigs remains poorly understood.

Therefore, the present study aimed to comprehensively investigate the effects of low-protein diets on finishing pigs using multi-omics approaches. By integrating data on growth performance, 16S rRNA sequencing, serum metabolomics, and muscle composition analysis, this study seeks to elucidate how LPDs affect gut microbiota, serum metabolic pathways, and muscle fatty acid profiles, ultimately providing more sustainable strategies for swine production.

## 2. Materials and Methods

All procedures involving pigs were carried out at Niu Jiaowan Pig Farm Co., Ltd. (Leizhou City, Zhanjiang, Guangdong Province, China) under the management of the Animal Welfare and Ethics Committee of China Agricultural University (Approval No. AW22302302-1-4).

### 2.1. Animals, Design and Management

A total of 180 healthy crossbred finishing pigs (Duroc × Liangguang Small Spotted; initial body weight 85.49 ± 4.90 kg) were enrolled in a 35-day trial and randomly assigned (six replicate pens per treatment, ten pigs per pen, male: female = 1:1) to one of three dietary regimens: Control (CON), receiving a standard protein diet (15.5% crude protein); Low-Protein 1 (LP1), containing 14.5% crude protein; or Low-Protein 2 (LP2), formulated with 13.5% crude protein. All diets supplied equivalent net energy, calcium, phosphorus and standardized ileal digestible amino acids (Table 1). The barn was equipped with an intelligent environmental control system capable of regulating temperature and humidity; however, during the experimental period, the indoor temperature remained above the preset range of 20 ± 2 °C due to the high ambient temperature in summer, while the relative humidity was maintained between 60% and 75%. The house floor was a partially slatted concrete floor. Each pen (3.2 m × 4.0 m) was equipped with a stainless-steel dry–wet feeder and a nipple-type automatic drinking system, allowing pigs ad libitum access to feed and water. In addition, vaccination was carried out according to the schedule detailed in Supplementary Table S1.

### 2.2. Sample Collection

Daily feed offered and refusals were recorded for each pen to compute average daily feed intake (ADFI). Body weights taken on experimental day 0 and day 35 were used to calculate average daily gain (ADG). Feed conversion ratio (FCR) was calculated as follows:

FCR = ADFI/ADG.

During days 33 to 35 of the experiment, fecal and feed samples (1 kg each) were collected from each pen after thoroughly cleaning the pen. Samples were collected twice daily (morning and evening), and care was taken to avoid fecal contamination. All samples were mixed thoroughly and dried in a 65 °C oven for 72 h. The dried feed and fecal samples were ground through a 1 mm sieve and held at 4 °C until analysis.

On the morning of day 35, after an overnight fast, one pig with an average body weight was selected from each replicate for blood sampling. Approximately 10 mL of blood was collected via the anterior vena cava using a sterile winged infusion set (22 G) connected to an EDTA-K_2_ anticoagulant tube (Shandong Ao Saite Medical Equipment, Heze, China) for hematological analysis, and another 10 mL was collected into vacuum tubes without anticoagulant for serum preparation. All blood samples were gently inverted several times immediately after collection and transported to the laboratory at 2–8 °C within 2 h. The anticoagulated blood was used for hematological determinations, while the non-anticoagulated blood was left standing for approximately 3 h at room temperature, followed by centrifugation at 3000× *g* for 10 min at 4 °C. The obtained serum was aliquoted into 2 mL cryotubes and stored at −80 °C until further biochemical and metabolomic analyses.

On day 36, following a 12 h fast, the same animals were transported (60–70 km/h for 1 h), allowed to rest for 4 h, then stunned electrically and slaughtered. Cecal contents were immediately collected and rapidly frozen (snap-frozen) in liquid nitrogen. Two samples of *Longissimus dorsi* (between the third and fourth last ribs on the left side) were excised—one kept on ice for meat-quality assays and the other frozen in liquid nitrogen before storage at −80 °C. Approximately 10 g each of liver tissue and mid-cecum chyme were collected from each pig as representative samples. About 1–2 g portions were subsampled into sterile 2 mL cryotubes, immediately frozen in liquid nitrogen, and stored at −80 °C until further analysis.

### 2.3. Chemical Analysis

#### 2.3.1. Nutrient Digestibility and Fecal Nitrogen Excretion

Dry matter (DM) and crude protein (CP) in feed and feces were analyzed using Association of Official Analytical Chemists [16] procedures; neutral detergent fiber (NDF) was determined on an ANKOM Fiber Analyzer (ANKOM Technology, Macedon, NY, USA) following Van Soest et al. [17]; gross energy (GE) was measured by an oxygen-bomb calorimeter (Model 6400, Parr Instrument Company, Moline, IL, USA) in accordance with ISO 9831:1998 [18]; and chromium concentrations were quantified by atomic absorption spectrophotometry (Z-5000; Hitachi, Tokyo, Japan).

Apparent total tract digestibility (ATTD) of each nutrient was then calculated as follows:

ATTD (%) = (1 – [DC × FN]/[FC × DN]) × 100

where DC is chromium concentration in the diet, FN is nutrient concentration in feces, FC is chromium concentration in feces, and DN is nutrient concentration in the diet.

The calculation of fecal nitrogen excretion per unit of body weight gain was performed according to the method described by Pan et al. [19], as follows:

Fecal nitrogen excretion per unit of body weight gain (g/kg) = nitrogen intake (g/day) ×  (1 − nitrogen digestibility)/average daily gain (kg/day).

#### 2.3.2. Hematological, Serum Biochemical, and Serum Metabolomics Analyses

Hematological parameters were determined using an automated hematology analyzer (ADVIA^®^ 2120i Hematology System, Siemens Healthcare Diagnostics Inc., Erlangen, Germany) according to the manufacturer’s instructions. The measured indicators included white blood cell count (WBC), red blood cell count (RBC), hemoglobin concentration (Hb), hematocrit (HCT), mean corpuscular volume (MCV), mean corpuscular hemoglobin (MCH), mean corpuscular hemoglobin concentration (MCHC), platelet count (PLT), red cell distribution width–standard deviation (RDW-SD), red cell distribution width–coefficient of variation (RDW-CV), and mean platelet volume (MPV).

Serum biochemical parameters were analyzed using a CX-4 automatic biochemical analyzer (Beckman Coulter, Brea, CA, USA). The measured items included alanine aminotransferase (ALT), aspartate aminotransferase (AST), alkaline phosphatase (ALP), total protein (TP), blood urea nitrogen (BUN), glucose (GLU), triglycerides (TG), and total cholesterol (TC). All analyses were performed using commercial assay kits (Nanjing Jiancheng Bioengineering Institute, Nanjing, China) according to the manufacturer’s protocols.

Non-targeted serum metabolomics analysis was performed according to Liu Et Al. [20]. In brief, serum (400 μL) was mixed 1:1 with methanol: acetonitrile (*v*/*v*), sonicated (40 kHz, 30 min, 5 °C), then centrifuged (13,000× *g*, 15 min, 4 °C). The supernatant was dried under nitrogen, reconstituted in acetonitrile: water (1:1, *v*/*v*), briefly sonicated, centrifuged again, and transferred to liquid chromatography–tandem mass spectrometry (LC-MS/MS) vials. Metabolites were profiled on a Thermo Fisher (Waltham, MA, USA) LC-MS/MS, annotated via Human Metabolome Database (HMDB), Metlin and Majorbio, and processed on the Majorbio Cloud Platform.

#### 2.3.3. Hepatic Biochemical Analyses

Hepatic antioxidant parameters, including superoxide dismutase (SOD) activity, total antioxidant capacity (T-AOC), and malondialdehyde (MDA) concentration, were determined using commercial assay kits, whereas inflammatory and stress-related indicators, including tumor necrosis factor-α (TNF-α), interleukin-6 (IL-6), interleukin-10 (IL-10), and heat shock protein 70 (HSP-70), were quantified using enzyme-linked immunosorbent assay (ELISA) kits (Nanjing Jiancheng Bioengineering Institute, Nanjing, China). Total protein concentration in the liver homogenates was determined using a bicinchoninic acid (BCA) protein assay kit from the same manufacturer. All hepatic parameters were normalized to total protein content and expressed per mg or g of total protein.

#### 2.3.4. Cecal Microbiota Analysis

Five cecal chyme samples (one per replicate) were processed for 16S rRNA profiling at Shanghai Majorbio. Genomic DNA was extracted (Omega Bio-tek, Norcross, GA, USA) and quantified by NanoDrop. The V3–V4 region was amplified with primers 338F/806R (Table S2), purified (AxyPrep), and measured on a Qubit 2.0 Fluorometer (Thermo Fisher Scientific, Waltham, MA, USA). Sequencing libraries were generated and run as 300 bp paired-end reads on an Illumina HiSeq PE300 platform (Illumina, San Diego, CA, USA). Raw reads were quality-filtered with Trimmomatic and merged using Fast Length Adjustment of Short reads (FLASH version 1.2.11) software, then clustered into operational taxonomic units (OTUs) at 97% similarity via a highly accurate method for generating OTU sequences from microbial amplicon reads. Taxonomic assignment employed the ribosomal database project database, and relative abundances were reported as percentages.

#### 2.3.5. Meat Quality Evaluation of *Longissimus dorsi* Muscle

Within 45 min post-slaughter, backfat thickness, muscle color, drip loss, pH_45min_ and loin eye area were assessed. Backfat was measured at the first rib, between the 6th–7th ribs and the last lumbar vertebra using a vernier caliper, and the mean of these three readings was recorded. The *Longissimus dorsi* cross-section’s width and height were also measured by caliper, with muscle color determined on the loin eye region using a CR-410 colorimeter (Konica Minolta, Tokyo, Japan). Loin eye area (cm^2^) was calculated as (width × height) × 0.7, and drip loss (%) was calculated as follows:

Drip loss (%) = [(initial muscle sample weight (g) − weight after 24 h suspension (g))/ initial muscle sample weight (g)] × 100.

#### 2.3.6. Amino Acid Composition of *Longissimus dorsi* Muscle

Freeze-dried *Longissimus dorsi* powder (0.1 g) was accurately weighed into ampoules, then hydrolyzed with 10 mL of 6 mol/L HCl at 110 ± 1 °C for 24 h. After cooling, the hydrolysate was transferred into a 100 mL volumetric flask, and 1 mL aliquots were evaporated to dryness at 60 °C. To remove residual acid, each dry residue was dissolved in 1 mL of distilled water and evaporated twice more. The final residue was reconstituted in 1 mL of double-distilled water, filtered through a 0.45 μm membrane, and 1 mL of filtrate was analyzed on a Hitachi L-8900 amino acid analyzer (Hitachi High-Technologies Corporation, Tokyo, Japan). Total amino acids were then quantified.

#### 2.3.7. Fatty Acid Profiling of *Longissimus dorsi* Muscle

Lipids were extracted from freeze-dried muscle samples using chloroform: methanol (2:1, *v*/*v*). Fatty acid methyl esters were prepared and analyzed by gas chromatography (Agilent 6890 series, Wilmington, DE, USA). Results were expressed as mg of fatty acid per g of dry-matter muscle.

### 2.4. Statistical Analysis

Except for microbiota and metabolomics data, all statistical analyses were performed using R software (version 4.3.2; R Foundation for Statistical Computing, Vienna, Austria). Each pen was considered the experimental unit. Data were tested for normality using the Shapiro–Wilk test and for homogeneity of variances using Levene’s test before performing ANOVA. Statistical analyses were performed using one-way ANOVA to compare treatment groups, followed by Duncan’s multiple range test for post hoc comparisons, with the Kruskal–Wallis rank—sum test applied when data failed parametric assumptions. Cecal microbiota differences and effect sizes were determined by LEfSe (LDA score ≥ 2.00), while serum metabolites showing FDR—adjusted *p* < 0.05 in one—way ANOVA were further evaluated by LDA (score ≥ 2.5) and subjected to both univariate and multivariate analyses in MetaboAnalyst 4.0 (The Metabolomics Innovation Centre, University of Alberta, Edmonton, Alberta, Canada). Trends were noted at 0.05 < *p* ≤ 0.10, significant differences at *p* ≤ 0.05, and highly significant differences at *p* ≤ 0.01.

## 3. Results

### 3.1. Environmental Parameters During the Experimental Period

Throughout the 35-day trial, the mean ambient temperature, relative humidity, and temperature–humidity index (THI) in the pig barn were recorded at 31.36 °C, 80.26%, and 85.14, respectively (Table 2).

### 3.2. Growth Performance

As shown in Table 3, no significant differences were observed in ADG, ADFI, or FCR across the three groups (*p* > 0.05). Pigs in all groups exhibited comparable final body weights, with mean ADG ranging from 542.09 to 559.82 g/d and FCR values between 4.06 and 4.19, suggesting that reducing dietary crude protein by up to 2% did not impair growth performance under the study conditions.

### 3.3. Nutrient Digestibility and Nitrogen Emission

The digestibility of DM, CP, GE, and NDF remained unaffected by dietary treatment (Table 4). In addition, compared to the control group, fecal nitrogen excretion per kilogram of weight gain decreased by 9% and 14% in the LP1 and LP2 groups, respectively (Table 5).

### 3.4. Hematological Parameters

RDW-SD was significantly lower in the LP1 and LP2 groups compared to the control group (*p* = 0.04; Table 6). No significant changes were observed in other indices, including WBC, RBC, Hb, or platelet-related parameters.

### 3.5. Serum Biochemical Parameters

Compared with the control group, pigs in both LP1 and LP2 groups exhibited a decreasing trend in BUN levels (*p* = 0.06), suggesting reduced nitrogen catabolism (Table 7). Other serum indicators, including ALT, AST, TP, glucose, triglycerides, and cholesterol, were not significantly affected by dietary protein levels.

### 3.6. Hepatic Antioxidant, Inflammatory, and Stress-Related Parameters

Hepatic antioxidant parameters, including SOD, T-AOC, and MDA, showed no significant differences among the three dietary groups. Similarly, hepatic concentrations of inflammatory and stress-related markers—TNF-α, IL-6, IL-10, and HSP-70—remained unaffected by protein reduction (Table 8).

### 3.7. Meat Quality Parameters

Backfat thickness was significantly greater in the LP1 group compared to both CON and LP2 groups (*p* < 0.01), whereas no significant differences were observed in other meat quality traits, including loin-eye area, muscle pH, color parameters (L*, a*, b*), or drip loss (Table 9).

### 3.8. Muscle Fatty Acid Composition

While no differences were observed in total fatty acids between CON and LP1 groups, pigs in the LP2 group exhibited significantly lower levels of several key fatty acids, including C12:0, C14:0, C16:0, C18:0, C18:1n9c, C20:0, C20:1, C22:0, C22:1n9 and total fatty acids (*p* < 0.05; Table 10).

### 3.9. Muscle Amino Acid Composition

Total amino acid concentrations were comparable among all groups; however, LP2-fed pigs showed significant reductions in most individual amino acids, including aspartic acid, glutamic acid, lysine, phenylalanine, and leucine, compared with the LP1 group (*p* < 0.05; Table 11). These changes were not observed in the LP1 group relative to CON.

### 3.10. Cecal Microbiota Composition and Differential Analysis

**Figure 1:**
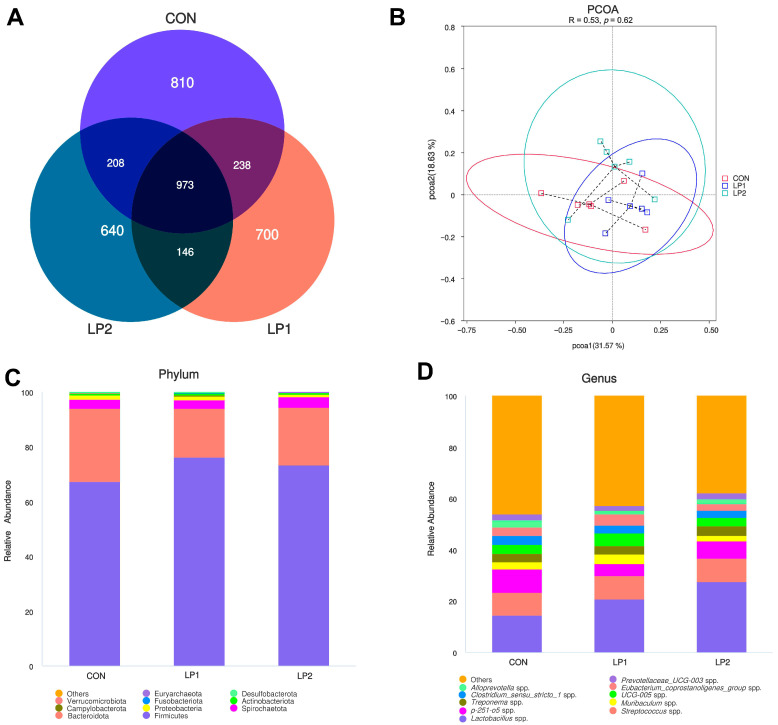
Effects of a low-protein diet on the cecal microbiota composition and structure of finishing pigs. (**A**) Venn diagram showing the shared and unique OTUs among treatments. (**B**) PCoA based on Bray–Curtis distances at the OTU level. (**C**) Relative abundance of bacterial phyla. (**D**) Relative abundance of bacterial genera. CON: standard diet (15.5% CP); LP1: reduced protein (14.5% CP); LP2: further reduction (13.5% CP).

To investigate microbial responses to dietary protein reduction, 16S rRNA sequencing was performed on cecal chyme. The α-diversity indices, including Chao1, Shannon, and Simpson (Table 12), revealed that pigs fed the LP2 diet exhibited significantly lower microbial richness and diversity compared with the CON group (*p* < 0.05), whereas the LP1 group showed intermediate values. A total of 973 OTUs were shared among all treatment groups, with CON, LP1, and LP2 harboring 810, 700, and 640 unique OTUs, respectively (Figure 1A). Despite these differences in richness, PCoA at the OTU level revealed no distinct clustering among the three dietary treatments (Figure 1B), suggesting similar overall microbial community structure.

**Figure 2:**
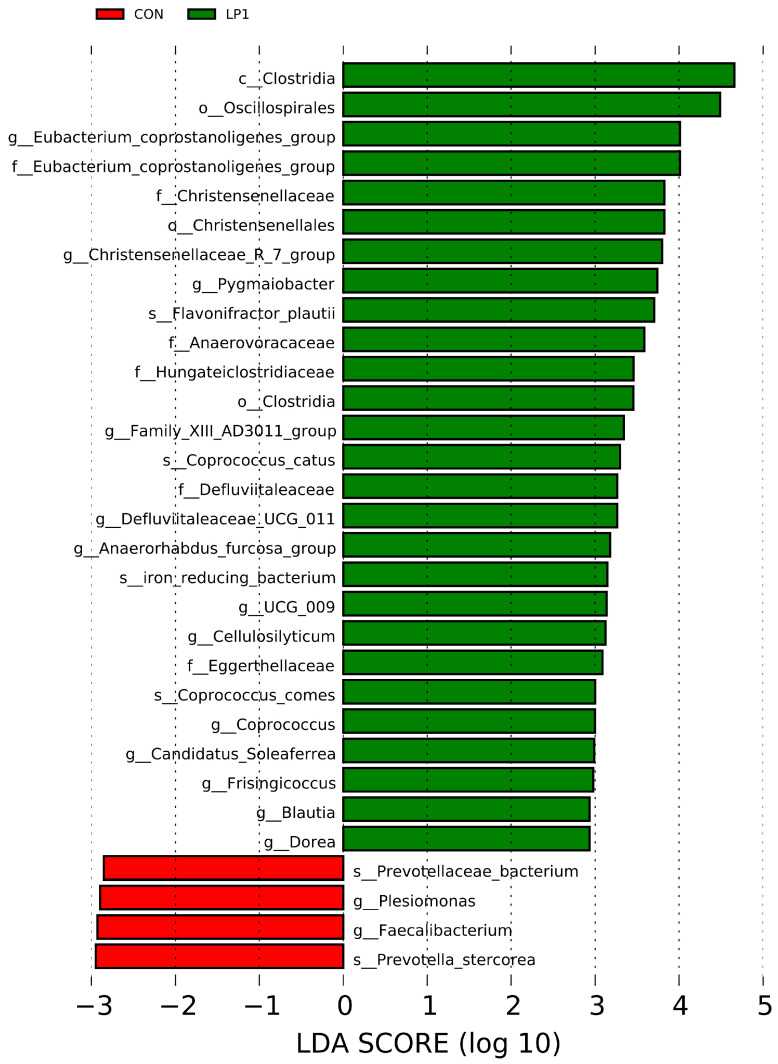
Differential bacterial taxa identified by LDA effect size among dietary treatments. The histogram shows the LDA scores of significantly different bacterial taxa (*p* < 0.05, LDA score > 2.50) among the three groups. Taxa with positive LDA scores are enriched in the indicated groups. CON: standard protein diet (15.5% CP); LP1: reduced protein diet (14.5% CP); LP2: further reduced protein diet (13.5% CP).

Microbial profiling at the phylum level identified Firmicutes, Bacteroidota, Spirochaetota, Proteobacteria, and Campylobacterota as the five most abundant taxa across all groups (Figure 1C). At the genus level, dominant microbes included *Prevotellaceae-UCG-003* spp., *Alloprevotella* spp., *Eubacterium-coprostanoligenes-group* spp., *Clostridium-sensu-stricto-1* spp., *UCG-005*, *Treponema* spp. (Figure 1D). However, LEfSe revealed distinct compositional differences among treatments (LDA > 2.50, *p* < 0.05). Specifically, the CON group was enriched in Prevotella_stercorea, *Faecalibacterium* spp., *Plesiomonas* spp., and Prevotellaceae bacterium, while LP1-fed pigs exhibited significantly higher abundances of beneficial taxa such as Clostridia, Oscillospirales, *Eubacterium_coprostanoligenes_group* spp., Christensenellaceae, and *Pygmaiobacter* spp. (Figure 2).

### 3.11. Serum Metabolomic Profiling

**Figure 3:**
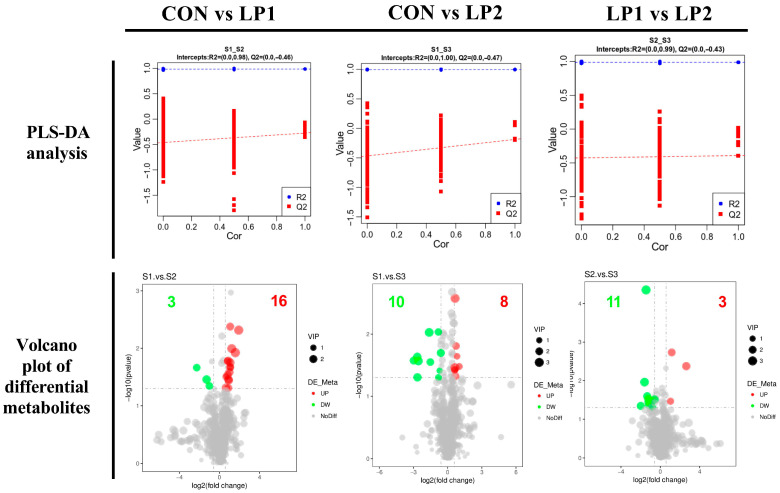
PLS-DA and Volcano plots of serum metabolites in finishing pigs fed diets with different protein levels. CON: standard diet (15.5% CP); LP1: reduced protein (14.5% CP); LP2: further reduction (13.5% CP).

To explore systemic metabolic alterations, we conducted non-targeted serum metabolomics and pathway enrichment analysis. PLS-DA demonstrated clear separation, indicating distinct metabolite signatures (*p* < 0.05; Figure 3). All metabolites with significant differences were listed in Table S3. Compared to the control, the LP1 group exhibited three upregulated metabolites, including PC (18:1/18:2), PE (18:0/22:5), and 2-Phenylethylamine, as well as sixteen downregulated serum metabolites. The LP2 group showed ten increased metabolites, such as PC (20:2/22:6) and PE (18:0/18:2), and eight decreased metabolites. Relative to LP1, LP2 pigs had eleven metabolites elevated, including PC (17:1/18:1) and PC (20:5e/22:5), and three reduced, reflecting a cumulative effect of further protein restriction.

**Figure 4:**
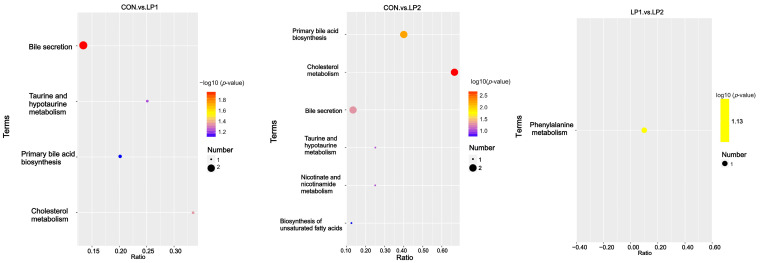
KEGG pathway enrichment analysis of differential serum metabolites in finishing pigs fed diets with different protein levels.

KEGG pathway analysis revealed that LP1 significantly suppressed pathways related to bile secretion and cholesterol metabolism (Figure 4). In LP2, these pathways were further inhibited, accompanied by downregulation of primary bile acid biosynthesis (*p* < 0.05). Moreover, LP2 displayed a marginal reduction in phenylalanine metabolism compared with LP1 (*p* = 0.07).

## 4. Discussion

Dietary optimization by partially substituting crude protein with crystalline amino acids such as lysine, tryptophan, and threonine has become a well-established nutritional approach to reduce dietary protein while maintaining performance [21]. In our study, both LP1 and LP2 diets, when supplemented with essential amino acids, supported growth performance comparable to the control group, consistent with the results of Han et al. [10], who observed that reducing dietary crude protein by 1–1.5% did not impair growth performance, nutrient digestibility, or meat quality in finishing pigs. By contrast, more drastic reductions without proper amino acid balancing have been reported to compromise intake and weight gain [22]. These findings collectively highlight that adequate amino acid supplementation is the key to sustaining animal performance under reduced protein regimens. In China, indigenous pig breeds such as Meishan, Fengjing, and Minzhu are known for their slower growth rates compared to commercial breeds like Duroc. Previous studies have reported that the ADG of these local breeds is significantly lower than that of Duroc pigs [23]. Most research on Chinese native pigs has focused on their reproductive performance and meat quality [24], with limited studies examining their growth parameters such as ADG and ADFI. Among the few studies on the Duroc × Liangguang Small Spotted crossbred, reports from domestic Chinese journals show that their ADG ranges from 488 to 549 g/d, ADFI from 2180 to 2350 g/d, and FCR from 4.15 to 4.76 during the finishing phase. These results align closely with those of our study, where the Duroc × Liangguang Small Spotted crossbred pigs exhibited ADG values ranging from 542.09 to 559.82 g/d, ADFI from 2263.71 to 2267.33 g/d, and FCR from 4.06 to 4.19. These results indicate that this hybrid cross, even with moderate dietary protein reduction, maintains favorable growth performance, supporting the notion that protein reduction with proper amino acid supplementation does not impair growth.

Beyond growth performance, the environmental benefits of low-protein diets deserve emphasis. Finishing pigs typically display low nutrient utilization efficiency, leading to excessive nitrogen excretion and environmental pollution [25]. Incorporating crystalline amino acids into reduced-protein diets enhances nitrogen utilization efficiency, thereby reducing nitrogen losses [4,5]. In the present trial, dietary protein reduction by 1–2% lowered nitrogen output per kilogram of weight gain by 9–14% without affecting apparent total tract digestibility, echoing previous reports that each 1% reduction in dietary protein reduces nitrogen excretion by 8–10% [11,12]. Thus, our results reinforce the environmental value of amino acid–fortified low-protein diets in reducing the ecological footprint of pig production.

Carcass traits and muscle nutrient composition further revealed threshold-dependent responses to protein reduction. LP1 pigs exhibited increased backfat thickness compared with both the control and LP2 groups, whereas no significant differences were observed in loin-eye area, drip loss, or pH values. Similar results have been reported by Morazán et al. [26], who found that reduced dietary protein increased backfat deposition, and by Madrid et al. [27], who suggested that decreased protein intake reduces the energy lost through protein catabolism and urinary nitrogen, thereby favoring lipid deposition. The absence of further fat accumulation in LP2 suggests that additional protein restriction may cross a metabolic threshold, limiting substrates for lipid accretion. Nutrient composition analyses of the *Longissimus dorsi* provided further evidence for these threshold-dependent effects. While total fatty acid levels did not differ between the control and LP1 groups, LP2 selectively reduced several key fatty acids, including palmitic acid (C16:0), stearic acid (C18:0), and oleic acid (C18:1n9c). These findings align with Liu et al. [28], who reported that reducing protein in Ningxiang pigs decreased the levels of C17:0, C17:1, and C18:3n3, and with Zhou et al. [29] and Teye et al. [30], who observed declines in saturated and unsaturated fatty acids under low-protein diets. Such changes are important because intramuscular fatty acid composition directly influences meat nutritional value and consumer preference. Similarly, the muscle amino acid profile showed that LP2-fed pigs had significantly reduced concentrations of both essential amino acids (e.g., lysine, phenylalanine, leucine) and flavor-related amino acids (e.g., glutamic acid, aspartic acid) compared with LP1. Essential amino acids are critical for protein deposition and growth [31], while glutamic and aspartic acids contribute to umami taste and pork palatability [32]. Comparable studies have reported minimal changes in pork amino acid composition under moderate protein restriction [33], whereas Berrazaga et al. [34] emphasized that protein source and amino acid balance shape muscle amino acid profiles. Together, these results suggest that moderate protein reduction (LP1) can maintain both nutritional and sensory qualities of pork, but further restriction (LP2) risks reducing the levels of amino acids central to meat quality.

Integration of microbiota and metabolomic data provides mechanistic insights into these observations. Excess dietary protein reaching the hindgut fosters proteolytic fermentation and favors pathogenic bacteria [35], whereas reducing protein intake or improving digestibility suppresses proteolytic fermentation and enriches beneficial taxa [36]. In the present study, the control group exhibited higher abundances of *Prevotella* spp. and *Faecalibacterium* spp., while LP1 enriched *Christensenellaceae* and *Clostridia*. These taxa have been linked with improved fiber fermentation and host lipid metabolism [15], suggesting that moderate protein reduction selectively reshaped the gut microbial community toward functions beneficial for energy utilization. The microbial shifts coincided with serum metabolomic changes, particularly the downregulation of bile secretion and cholesterol metabolism pathways. Such alterations are consistent with Xu et al. [7], who reported reduced bile acid synthesis and altered lipid metabolism in pigs fed long-term low-protein diets. Moreover, the metabolic reprogramming observed here coincided with differences in carcass fatness and muscle nutrient composition, supporting the notion that gut microbiota and host metabolism jointly mediate the effects of dietary protein reduction. Supporting this, Gurr et al. [37] demonstrated that low-protein diets increased plasma triiodothyronine levels, indicating enhanced lipid metabolism, and Fuller [38] proposed that protein restriction shifts metabolic resources from protein synthesis toward lipid storage. Thus, the combined evidence suggests that dietary protein reduction regulates a diet–microbiota–host axis, which governs lipid turnover, nitrogen utilization, and muscle nutrient deposition. In addition, considering that the mean barn temperature during the trial reached 31.36 °C, pigs in this study were exposed to heat stress conditions. Heat stress has been shown to impair intestinal integrity, alter microbial composition, and reduce feed intake and growth performance [39]. Previous studies have reported that low-protein diets can alleviate heat stress by reducing metabolic heat production, improving nitrogen utilization efficiency, and maintaining antioxidant balance [40,41]. Therefore, the beneficial microbial and metabolic adaptations observed here may partly reflect the mitigating effects of low-protein diets under heat-stress conditions. Future research should further explore the interaction between dietary protein levels and heat stress responses to better understand their combined influence on intestinal health and metabolism.

## 5. Conclusions

This study demonstrates that a moderate reduction in dietary crude protein by approximately 1–2% (equivalent to 6.4–12.9% relative decrease in total protein content), with essential amino acid supplementation, maintains growth performance and nutrient digestibility in finishing pigs. While backfat thickness increased under moderate restriction, excessive protein reduction led to decreased levels of key muscle fatty acids and amino acids, which may negatively affect meat quality. Additionally, low-protein diets altered the cecal microbiota, enriching beneficial taxa such as *Clostridia* and *Christensenellaceae*, and modulated serum metabolic pathways, including the downregulation of bile secretion and cholesterol metabolism. These findings highlight the influence of dietary protein levels on host–microbiota–metabolite interactions and support the application of precision protein nutrition strategies to optimize carcass traits and metabolic responses. Further research is warranted to evaluate the long-term physiological and microbial impacts of protein-restricted feeding in practical swine production systems.

## Figures and Tables

**Table 1:** Composition and nutrient levels of experimental diets (%, as-fed basis).

Ingredients	Treatments ^1^		
	CON	LP1	LP2
Corn	66.04	67.84	69.16
Soybean meal	20.80	17.40	13.70
Sorghum	3.00	3.00	3.00
Wheat bran	3.50	3.50	3.50
Rice bran meal	3.50	4.80	6.90
Limestone	1.22	1.25	1.28
Dicalcium phosphate	0.37	0.36	0.33
Sodium bicarbonate	0.15	0.15	0.15
Salt	0.38	0.38	0.38
L-lysine HCl (70%)	0.27	0.43	0.60
DL-Methionine (98.5%)	0.02	0.03	0.05
L-Threonine (98.5%)	0.00	0.05	0.09
L-Tryptophan (25%)	0.00	0.06	0.11
Chromium oxide	0.25	0.25	0.25
Premix ^2^	0.50	0.50	0.50
Nutrient levels ^3^			
Digestible energy, Kcal/kg	3272	3254	3227
Net energy, Kcal/kg	2370	2381	2386
Crude protein	15.50	14.53	13.53
Calcium	0.62	0.62	0.61
Digestible phosphorus	0.17	0.17	0.17
Lysine	0.97	0.97	0.96
Methionine	0.29	0.29	0.29
Threonine	0.61	0.61	0.60
Tryptophan	0.17	0.17	0.17
SID Lysine	0.83	0.83	0.83
SID Methionine	0.26	0.26	0.26
SID Threonine	0.49	0.49	0.49
SID Tryptophan	0.13	0.13	0.13

^1^ CON: normal protein diet with 15.5% crude protein; LP1: low-protein diet 1 with 14.5% crude protein; LP2: low-protein diet 2 with 13.5% crude protein. ^2^ Premix provided the following per kg of diet: vitamin A, 12,000 IU; vitamin E, 30 IU; vitamin D3, 2500 IU; vitamin K3, 30 mg; vitamin B1, 1.5 mg; vitamin B12, 12 μg; vitamin B6, 3 mg; pantothenic acid, 15 mg; riboflavin, 4 mg; choline chloride, 400 mg; niacin, 40 mg; biotin, 0.1 mg; folic acid, 0.7 mg; Fe, 90 mg; Mn, 40 mg; Cu, 8.8 mg; Zn, 100 mg; Se, 0.3 mg; I, 0.35 mg. ^3^ Nutrient levels were calculated values. SID: Standard ileal digestible.

**Table 2:** Temperature, humidity, and temperature and humidity index of the pig house during the experimental period.

Items	Temperature, °C	Relative Humidity, %	Temperature–Humidity Index
Mean	31.36	80.26	85.14

**Table 3:** Effects of a low-protein diet on growth performance of finishing pigs.

Items ^1^	Treatments ^2^			SEM ^3^	*p*-Value
	CON	LP1	LP2		
IBW, kg	85.48	85.49	85.49	0.03	0.93
FBW, kg	104.45	105.09	104.61	0.33	0.40
ADG, g/d	542.09	559.82	546.22	9.71	0.43
ADFI, g/d	2263.71	2267.33	2264.33	11.65	0.97
FCR, g/g	4.19	4.06	4.16	0.08	0.46

^1^ IBW: initial body weight; FBW: final body weight; ADG: average daily gain; ADFI: average daily feed intake; FCR: feed conversion ratio. ^2^ CON: normal protein diet with 15.5% crude protein; LP1: low-protein diet 1 with 14.5% crude protein; LP2: low-protein diet with 13.5% crude protein. ^3^ SEM is the standard error of the mean.

**Table 4:** Effects of a low-protein diet on apparent total tract digestibility of nutrients in finishing pigs (%).

Items ^1^	Treatments ^2^			SEM ^3^	*p*-Value
	CON	LP1	LP2		
DM	86.30	85.83	86.28	0.47	0.74
CP	83.48	82.02	82.89	0.70	0.37
GE	87.32	86.94	87.52	0.42	0.63
NDF	43.40	45.83	37.19	3.47	0.24

^1^ DM: dry matter; CP: crude protein; GE: gross energy; NDF: neutral detergent fiber. ^2^ CON: standard diet (15.5% CP); LP1: reduced protein (14.5% CP); LP2: further reduction (13.5% CP). ^3^ SEM: standard error of the mean.

**Table 5:** Effects of a low-protein diet on nitrogen emission per kilogram of weight gain in finishing pigs.

Items	Treatments ^1^			SEM ^2^	*p*-Value
	CON	LP1	LP2		
Nitrogen emission per kg of weight gain	131	119	113	0.12	0.63

^1^ CON: standard diet (15.5% CP); LP1: reduced protein (14.5% CP); LP2: further reduction (13.5% CP). ^2^ SEM is the standard error of the mean.

**Table 6:** Effects of a low-protein diet on hematological parameters of finishing pigs.

Items ^1^	Treatments ^2^			SEM ^3^	*p*-Value
	CON	LP1	LP2		
WBC, 10^9^/L	15.45	15.12	17.18	1.25	0.48
RBC, 10^12^/L	7.16	7.31	6.84	0.43	0.75
Hb, g/L	128.10	131.55	119.44	7.27	0.50
HCT, %	45.41	46.51	41.96	2.51	0.44
MCV, fL	63.50	63.95	61.64	0.87	0.17
MCH, pg	17.88	18.08	17.56	0.29	0.44
MCHC, g/L	281.80	282.64	284.67	2.74	0.76
PLT, 10^9^/L	361.40	335.55	327.33	31.77	0.74
RDW-SD, fL	43.97 ^a^	41.04 ^b^	40.01 ^b^	1.08	0.04
RDW-CV, %	20.11	18.89	19.07	0.42	0.10
MPV, fL	11.83	11.93	12.21	0.38	0.78
P-LCR, %	44.12	45.57	48.66	3.34	0.64
PCT, %	0.38	0.35	0.32	0.04	0.57

^1^ WBC: white blood cells; RBC: red blood cells; Hb: hemoglobin; HCT: hematocrit; MCV: mean corpuscular volume; MCH: mean corpuscular hemoglobin; MCHC: mean corpuscular hemoglobin concentration; PLT: platelet count; RDW-SD: red cell distribution width-standard deviation; RDW-CV: red cell distribution width-coefficient of variation; MPV: mean platelet volume; P-LCR: platelet large cell ratio; PCT: procalcitonin. ^2^ CON: standard diet (15.5% CP); LP1: reduced protein (14.5% CP); LP2: further reduction (13.5% CP). ^3^ SEM, standard error of the mean. ^a,b^ Different superscripts within a row indicate a significant difference, *p* < 0.05.

**Table 7:** Effects of a low-protein diet on serum biochemical parameters of finishing pigs.

Items ^1^	Treatments ^2^			SEM ^3^	*p*-Value
	CON	LP1	LP2		
ALT, U/L	42.92	40.08	45.50	2.10	0.20
AST, U/L	43.83	33.42	40.00	5.83	0.45
ALP, IU/L	137.58	157.92	154.92	17.42	0.68
TP, g/L	68.45	68.38	67.80	0.96	0.87
BUN, mmol/L	3.09	2.87	2.44	0.19	0.06
GLU, mmol/L	3.99	3.72	3.64	0.15	0.24
TG, mmol/L	0.40	0.45	0.45	0.03	0.53
TC, mmol/L	2.40	2.67	2.62	0.12	0.27

^1^ ALT: alanine aminotransferase; AST: aspartate aminotransferase; ALP: alkaline phosphatase; TP: total protein; BUN: blood urea nitrogen; GLU: glucose; TG: total triglycerides; TC: total cholesterol. ^2^ CON: standard diet (15.5% CP); LP1: reduced protein (14.5% CP); LP2: further reduction (13.5% CP). ^3^ SEM is the standard error of the mean.

**Table 8:** Effects of a low-protein diet on liver antioxidants, inflammatory, and stress-related indicators of finishing pigs.

Items ^1^	Treatments ^2^			SEM ^3^	*p*-Value
	CON	LP1	LP2		
SOD, U/mg	266.07	229.11	278.73	21.19	0.28
T-AOC, mmol/g	1.61	1.34	1.50	0.17	0.56
MDA, nmol/mg	3.82	2.59	3.73	0.43	0.13
TNF-α, pg/mg	24.53	18.10	22.99	2.42	0.20
IL-6, pg/mg	33.75	29.19	34.81	2.18	0.46
IL-10, pg/mg	15.82	11.59	16.81	2.56	0.35
HSP-70, ng/mg	67.66	62.46	71.16	8.02	0.75

^1^ SOD: superoxide dismutase; T-AOC: total antioxidant capacity; MDA: malondialdehyde; TNF-α: tumor necrosis factor-α; IL-6: interleukin-6; IL-10: interleukin-10; HSP-70: heat shock protein-70. ^2^ CON: standard diet (15.5% CP); LP1: reduced protein (14.5% CP); LP2: further reduction (13.5% CP). ^3^ SEM is the standard error of the mean.

**Table 9:** Effects of a low-protein diet on meat quality of finishing pigs.

Items ^1^	Treatments ^2^			SEM ^3^	*p*-Value
	CON	LP1	LP2		
BW before slaughter, kg	113.21	113.49	111.96	1.08	0.54
Loin-eye area, cm^2^	34.85	29.71	28.81	2.18	0.18
pH_45min_	6.16	6.09	6.08	0.06	0.68
pH_24h_	5.74	5.69	5.77	0.04	0.43
ΔpH (pH_45min_–pH_24h_)	0.43	0.40	0.31	0.05	0.74
L*	41.54	42.07	42.43	1.24	0.88
a*	2.76	3.33	3.01	0.33	0.51
b*	3.15	5.10	3.28	1.10	0.38
Backfat thickness, mm	34.44 ^b^	39.76 ^a^	35.31 ^b^	1.22	<0.01
Drip loss, %	3.16	3.03	3.02	0.01	0.08

^1^ BW: body weight; pH_45min_: pH value at 45 min post-slaughter; pH_24h_: pH value at 24 h post-slaughter. L*: lightness; a*: redness; b*: yellowness. ^2^ CON: standard diet (15.5% CP); LP1: reduced protein (14.5% CP); LP2: further reduction (13.5% CP). ^3^ SEM: standard error of the mean. ^a,b^ Different superscripts within a row indicate a significant difference, *p* < 0.05.

**Table 10:** Effects of a low-protein diet on muscle fatty acids of finishing pigs (dry matter, g/kg).

Items	Treatments ^1^			SEM ^2^	*p*-Value
	CON	LP1	LP2		
C12:0	0.13 ^ab^	0.16 ^a^	0.11 ^b^	0.01	0.04
C14:0	2.27 ^ab^	2.84 ^a^	1.92 ^b^	0.24	0.03
C14:1	0.04	0.05	0.03	0.01	0.10
C15:0	0.03	0.04	0.03	0.01	0.08
C16:0	40.10 ^ab^	50.78 ^a^	34.64 ^b^	4.25	0.03
C16:1	5.37	6.46	4.57	0.58	0.08
C17:0	0.18	0.21	0.15	0.02	0.07
C18:0	21.06 ^ab^	27.77 ^a^	19.06 ^b^	2.22	0.02
C18:1n9c	63.07 ^ab^	81.60 ^a^	54.81 ^b^	7.31	0.04
C18:2n6c	10.39	11.99	9.33	1.08	0.23
C18:3n3	0.35	0.39	0.29	0.04	0.20
C20:0	0.45 ^ab^	0.59 ^a^	0.41 ^b^	0.05	0.04
C20:1	1.41 ^ab^	1.83 ^a^	1.14 ^b^	0.18	0.03
C21:0	0.54	0.62	0.45	0.06	0.18
C20:2	0.07	0.08	0.06	0.01	0.19
C20:3n6	0.24	0.29	0.26	0.01	0.10
C20:4n6	1.42	1.56	1.52	0.09	0.54
C20:3n3	0.08	0.10	0.06	0.01	0.07
C22:0	0.06 ^b^	0.08 ^a^	0.06 ^b^	0.01	<0.01
C20:5n3	0.03 ^b^	0.04 ^a^	0.04 ^ab^	0.01	0.02
C22:1n9	0.07 ^ab^	0.09 ^a^	0.06 ^b^	0.01	<0.01
C23:0	0.03	0.03	0.03	0.002	0.04
C24:0	0.06	0.07	0.05	0.003	0.05
C24:1	0.05	0.05	0.05	0.003	0.09
C22:6n3	0.04	0.05	0.05	0.005	0.19
Total fatty acids	147.54 ^ab^	187.77 ^a^	129.17 ^b^	15.76	0.04

^1^ CON: standard diet (15.5% CP); LP1: reduced protein (14.5% CP); LP2: further reduction (13.5% CP). ^2^ SEM: standard error of the mean. ^a,b^ Different superscripts within a row indicate a significant difference, *p* < 0.05.

**Table 11:** Effects of a low-protein diet on muscle amino acid of finishing pigs (%).

Items	Treatments ^1^			SEM ^2^	*p*-Value
	CON	LP1	LP2		
Aspartic acid	6.72 ^ab^	6.48 ^b^	7.13 ^a^	0.16	0.02
Threonine	3.31 ^ab^	3.19 ^b^	3.50 ^a^	0.08	0.03
Serine	2.82 ^ab^	2.71 ^b^	2.98 ^a^	0.06	0.02
Glutamic acid	11.10 ^ab^	10.72 ^b^	11.71 ^a^	0.25	0.03
Proline	2.51 ^ab^	2.41 ^b^	2.60 ^a^	0.05	0.03
Glycine	3.06 ^ab^	2.92 ^b^	3.14 ^a^	0.06	0.03
Alanine	4.04 ^ab^	3.89 ^b^	4.25 ^a^	0.08	0.02
Cysteine	0.82 ^ab^	0.77 ^b^	0.86 ^a^	0.02	0.01
Valine	3.65 ^ab^	3.53 ^b^	3.88 ^a^	0.09	0.03
Methionine	2.04 ^ab^	1.92 ^b^	2.17 ^a^	0.05	<0.01
Isoleucine	3.46 ^ab^	3.35 ^b^	3.68 ^a^	0.08	0.03
Leucine	5.79 ^ab^	5.60 ^b^	6.14 ^a^	0.14	0.03
Tyrosine	2.40 ^ab^	2.31 ^b^	2.55 ^a^	0.06	0.02
Phenylalanine	2.79 ^ab^	2.68 ^b^	2.95 ^a^	0.07	0.03
Histidine	3.25 ^ab^	3.13 ^b^	3.51 ^a^	0.09	0.02
Lysine	6.50 ^ab^	6.29 ^b^	6.90 ^a^	0.15	0.02
Arginine	4.56 ^ab^	4.40 ^b^	4.80 ^a^	0.10	0.02

^1^ CON: standard diet (15.5% CP); LP1: reduced protein (14.5% CP); LP2: further reduction (13.5% CP). ^2^ SEM: standard error of the mean. ^a,b^ Different superscripts within a row indicate a significant difference, *p* < 0.05.

**Table 12:** Effect of a low-protein diet on the α-diversity in feces of finishing pigs.

Items	Treatments ^1^			SEM ^2^	*p*-Value
	CON	LP1	LP2		
Chao1	891.32 ^a^	857.12 ^ab^	780.30 ^b^	26.43	0.05
Shannon	7.56 ^a^	7.19 ^ab^	6.61 ^b^	0.23	0.05
Simpson	0.97 ^a^	0.96 ^a^	0.92 ^b^	0.01	0.02

^1^ CON: standard diet (15.5% CP); LP1: reduced protein (14.5% CP); LP2: further reduction (13.5% CP). ^2^ SEM: standard error of the mean. ^a,b^ Different superscripts within a row indicate a significant difference, *p* < 0.05.

## Data Availability

The original data of this study is included in the article and further information is available upon reasonable request to the corresponding author.

## Supplementary Materials

The following supporting information can be downloaded at https://www.mdpi.com/article/10.3390/ani15223234/s1. Table S1: Vaccination schedule of experimental pigs. Table S2: Primer sequences used for 16S rRNA gene amplification. Table S3: Significantly differential metabolites in serum among LP1, LP2, and control groups.
