# Supplementary material for: Moderate Reduction in Dietary Protein Improves Muscle Composition and Modulates Gut Microbiota and Serum Metabolome Without Compromising Growth in Finishing Pigs
Source: Animals (Basel). 2025 Nov 7;15(22):3234. doi: 10.3390/ani15223234 (PMC12649659; doi:10.3390/ani15223234)
Supplement: Supplementary file 1 [file animals-15-03234-s001.zip › animals-3925942-supplementary.pdf]

Table S1 Vaccination schedule of experimental pigs

Day(s) Post-Weaning	Vaccine Type	Route of Administration
Day 3	Pseudorabies (Intranasal)	Intranasal
Day 14	Porcine Circovirus Type 2 (PCV2)	Intramuscular
Day 21	Classical Swine Fever Live Vaccine	Intramuscular
Day 28	Mycoplasmal Pneumonia	Intramuscular
Day 35	Pseudorabies (Booster)	Intramuscular
Day 45	Foot-and-Mouth Disease (FMD), Primary Immunization (Serotypes O & A)	Intramuscular
Day 65	Classical Swine Fever (Booster)	Intramuscular
Day 75	Foot-and-Mouth Disease (FMD, Serotypes O & A) (Booster)	Intramuscular
Day 90	Porcine Reproductive and Respiratory Syndrome (PRRS) (Optional)	Intramuscular

Table S2. Primer sequences used for 16S rRNA gene amplification.

Primer	Sequence (5′–3′)	Target region
338F	ACTCCTACGGGAGGCAGCA	16S rRNA V3–V4
806R	GGACTACHVGGGTWTCTAAT	16S rRNA V3–V4

Table S3. Significantly differential metabolites in serum among LP1, LP2, and control groups

Comparison	Metabolite	Fold Change	*p*-value	VIP	Up_Down
LP1.vs.LP2	PC (18:1/18:2)	0.37	<0.01	3.08	down
LP1.vs.LP2	PC (20:2/22:6)	2.24	<0.01	2.09	up
LP1.vs.LP2	PE (18:0/22:5)	6.23	<0.01	2.41	up
LP1.vs.LP2	PC (17:1/18:1)	0.33	0.01	2.75	down
LP1.vs.LP2	PC (16:0/17:1)	0.40	0.03	2.30	down
LP1.vs.LP2	PC (19:2/18:5)	0.43	0.03	2.28	down
LP1.vs.LP2	PC (20:5e/22:5)	0.66	0.03	2.43	down
LP1.vs.LP2	PE (18:0/18:2)	0.57	0.03	2.20	down
LP1.vs.LP2	2-Phenylethylamine	2.05	0.03	1.60	up
LP1.vs.LP2	DL-Dipalmitoylphosphatidylcholine	0.43	0.04	2.18	down
LP1.vs.LP2	10-Hydroxydecanoic acid	0.52	0.04	1.90	down
LP1.vs.LP2	Prostaglandin E1	0.44	0.04	2.09	down
LP1.vs.LP2	12-epi Leukotriene B4	0.25	0.05	2.29	down
LP1.vs.LP2	PC (19:2/18:3)	0.58	0.05	1.75	down
CON.vs.LP1	PC (16:0/17:1)	2.10	<0.01	1.96	up

CON.vs.LP1	PC (16:1/17:2)	3.78	<0.01	2.88	up
CON.vs.LP1	PE (16:0/18:0)	2.34	0.01	2.72	up
CON.vs.LP1	DL-Dipalmitoylphosphatidylcholine	3.00	0.01	2.96	up
CON.vs.LP1	PC (17:1/18:2)	1.76	0.02	1.35	up
CON.vs.LP1	SM (d18:1/18:0)	1.82	0.02	2.55	up
CON.vs.LP1	PC (15:0/18:1)	2.11	0.02	2.32	up
CON.vs.LP1	Lithocholic Acid	2.13	0.02	1.69	up
CON.vs.LP1	PC (17:1/18:1)	2.08	0.02	1.91	up
CON.vs.LP1	Taurocholic acid	0.21	0.02	1.72	down
CON.vs.LP1	PC (19:2/18:3)	2.00	0.03	2.11	up
CON.vs.LP1	PC (16:0/18:2)	1.75	0.03	2.20	up
CON.vs.LP1	PC (18:1/18:2)	1.55	0.03	1.28	up
CON.vs.LP1	OxPC (18:0-20:3+1O(1Cyc))	1.86	0.03	2.30	up
CON.vs.LP1	LPA 22:6	0.41	0.03	2.36	down
CON.vs.LP1	PC (16:0/16:0)	1.91	0.04	2.16	up
CON.vs.LP1	Mupirocin	0.50	0.05	1.76	down
CON.vs.LP1	Dibutyl sebacate	1.56	0.05	2.25	up
CON.vs.LP1	PC (17:0/18:2)	1.85	0.05	1.89	up
CON.vs.LP2	P-Aminohippuric Acid	1.57	<0.01	3.02	up
CON.vs.LP2	PC (18:1/18:2)	0.57	0.01	1.80	down
CON.vs.LP2	Prostaglandin E1	0.32	0.01	2.84	down
CON.vs.LP2	(±)12(13)-DiHOME	1.66	0.02	1.90	up
CON.vs.LP2	N1-{3-[2-(2-pyridyl)ethyl]phenyl}-4-chlorobenzene-1-sulfonamide	0.66	0.02	2.29	down
CON.vs.LP2	Methyltestosterone	1.76	0.02	1.26	up
CON.vs.LP2	Glycocholic acid	0.16	0.02	2.53	down
CON.vs.LP2	Taurocholic acid	0.13	0.03	2.18	down
CON.vs.LP2	12-epi Leukotriene B4	0.17	0.03	2.77	down
CON.vs.LP2	Asaraldehyde	0.35	0.03	1.97	down
CON.vs.LP2	PC (16:1/17:2)	2.02	0.03	1.45	up
CON.vs.LP2	2-Hydroxyphenylalanine	1.55	0.03	3.06	up
CON.vs.LP2	Nicotinuric Acid	1.54	0.04	1.73	up
CON.vs.LP2	PC (19:0/20:4)	1.53	0.04	1.43	up
CON.vs.LP2	N2-tetrahydrofuran-2-ylmethyl-4-(4-fluorophenyl)-1,3-thiazol-2-amine	0.61	0.04	1.15	down
CON.vs.LP2	Docosapentaenoic acid	1.60	0.05	2.16	up
CON.vs.LP2	PC (16:0e/17:0)	0.16	0.05	2.50	down
CON.vs.LP2	Miquelianin	0.57	0.05	1.42	down
